# Capturing the Two Dimensions of Residential Segregation at the Neighborhood Level for Health Research

**DOI:** 10.3389/fpubh.2014.00118

**Published:** 2014-08-25

**Authors:** Masayoshi Oka, David W. S. Wong

**Affiliations:** ^1^Division of Public Health Sciences, Department of Surgery, School of Medicine, Washington University in St. Louis, St. Louis, MO, USA; ^2^Division of Epidemiology and Public Health, School of Medicine, University of Alcalá, Alcalá de Henares, Spain; ^3^Department of Geography and GeoInformation Science, College of Science, George Mason University, Fairfax, VA, USA; ^4^Department of Geography, University of Hong Kong, Pokfulam, Hong Kong

**Keywords:** residential segregation, local spatial entropy-based diversity index, local spatial isolation index, racial/ethnicity segregation, socioeconomic segregation

## Abstract

Two conceptual and methodological foundations of segregation studies are that (i) segregation involves more than one group, and (ii) segregation measures need to quantify how different population groups are distributed across space. Therefore, percentage of population belonging to a group is not an appropriate measure of segregation because it does not describe how populations are spread across different areal units or neighborhoods. In principle, evenness and isolation are the two distinct dimensions of segregation that capture the spatial patterns of population groups. To portray people’s daily environment more accurately, segregation measures need to account for the spatial relationships between areal units and to reflect the situations at the neighborhood scale. For these reasons, the use of local spatial entropy-based diversity index (*SH_i_*) and local spatial isolation index (*S_i_*) to capture the evenness and isolation dimensions of segregation, respectively, are preferable. However, these two local spatial segregation indexes have rarely been incorporated into health research. Rather ineffective and insufficient segregation measures have been used in previous studies. Hence, this paper empirically demonstrates how the two measures can reflect the two distinct dimensions of segregation at the neighborhood level, and argues conceptually and set the stage for their future use to effectively and meaningfully examine the relationships between residential segregation and health.

## Introduction

In the United States (US), residential segregation has long been considered to shape health (i.e., health behaviors and health outcomes) as blacks and/or poor individuals are not distributed across geographic locations in the same manners as other groups (1–3). Note that all racial groups in this paper refer to non-Hispanic populations. A recent review article shows a growing number of US studies focusing on such concerns (4). However, the conceptual and methodological inconsistencies across previous studies limit the ability to draw specific conclusions about the relationships between residential segregation and health.

For instance, previous studies (4) have adopted either a global or local approach in measuring segregation. The former approach is based on the use of global measures that summarize the condition of a county or metropolitan area as a whole; these measures are used for inter-city, inter-regional, or inter-metropolitan comparisons. On the other hand, the latter approach is based on the use of local measures derived from data for US census tracts or block groups (sometimes zip-code areas); these measures are used for neighborhood comparisons. While these two approaches have different purposes, they also have different degrees of relevance to health research. Known as the modifiable areal unit problem (5), or more narrowly the issue of geographic scale, segregation levels vary between counties and metropolitan areas partly because of their different sizes (6, 7). Thus, scale effect obscures inter-city, inter-regional, or inter-metropolitan comparisons. More importantly, a global approach fails to recognize the important variations of segregation levels across local areas or neighborhoods [e.g., Ref. (8–10)]. Unlike other areal units, the US census tracts are designed to be relatively homogeneous in regard to population characteristics, economic status, and living conditions (11). They are also delineated historically in accordance with uniform standards (12). Using them as local units (i.e., for the local approach) to examine the relationships between residential segregation and health is appropriate as they can portray people’s daily environment more accurately than using other areal unit entities.

**Figure 1:**
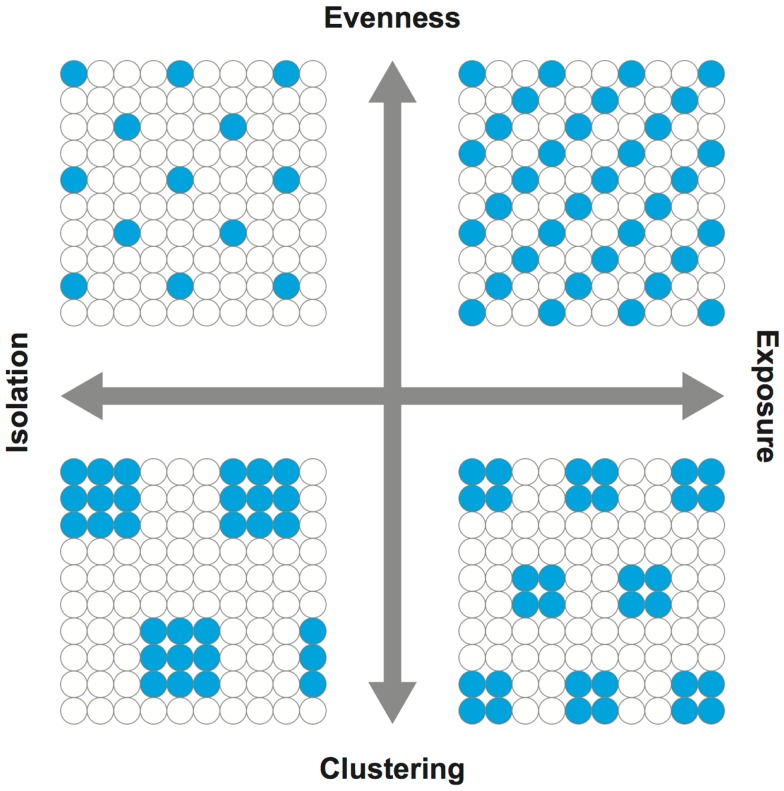
**Dimensions of segregation**. Adopted from Reardon and O’Sullivan (17). In capturing the spatial patterns of population groups, evenness is the opposite of clustering while isolation is the opposite of exposure. In simplest forms, the upper right quadrant may be conceived as a possible scenario for integration or diversity, whereas the lower left quadrant for isolation.

Equally important to the choice of areal units (i.e., the scale of analysis), the use of effective and meaningful segregation measures is critical in segregation studies. More than two decades ago, Massey and Denton (13) conducted an extensive and in-depth literature review on various segregation measures and classified them into five dimensions: (i) evenness (the differential distribution of population groups), (ii) exposure or, its counterpart, isolation (the potential interaction of population groups), (iii) concentration, similar to the concept of density (the distributional intensity of population groups), (iv) centralization (the dispersion of population groups with respect to the city center), and (v) clustering (the degree of spatial separation or proximity of population groups). Because segregation measures without considering the spatial relationships are not effective [e.g., Ref. (14–16)], however, Reardon and O’Sullivan (17) concluded that evenness and isolation are the two distinct conceptual dimensions of segregation (Figure 1). Here, the centralization and concentration dimensions were omitted from the conceptual framework since both are regarded as specific subcategories of the evenness dimension (17). Johnston et al. (18) also concluded that the five conceptual dimensions of segregation should be reduced to two (i.e., evenness and isolation); they were unable to replicate Massey and Denton’s empirical work in identifying the original five dimensions using the 1980–2000 US censuses. The dimensions of evenness and isolation are generally regarded to be distinctive, and therefore, segregation measures reflecting these two dimensions are preferable. By incorporating the concept from spatial statistics (19, 20), Wong (9, 10) developed a family of local spatial segregation indexes to capture the evenness and isolation dimensions of segregation at the neighborhood level.

Despite the conceptual importance of the local approach and thus the use of local spatial segregation indexes, they have rarely been incorporated into health research. A disconnect between the concept and measurement of segregation and its applications to health research would undermine the study of the role of residential segregation in health. In reference to the recent review article (4), for example, most previous studies based on a local approach have used the percentage of population belonging to a group (e.g., percent white and black) as a measure of segregation; among them, a large body of literature has focused on adult mortality and pregnancy outcomes (e.g., low birth weight and/or preterm birth). Possibly, due to the adoptions of ineffective and insufficient segregation measures, the associations of residential segregation with adult morality (21–26) and pregnancy outcomes (27–32) have been mixed. Drawing from a rather inconclusive body of literature (including the 12 studies listed above) nevertheless, Kramer and Hogue (4) suggested that the clustering (i.e., unevenness) and isolation dimensions of segregation may have a protective and adverse effect on health, respectively. While White and Borrell (33) conceived percentages as a “proxy” measure of segregation, percentages are not a measure of segregation because they cannot quantify how different population groups are distributed across space. Of particular importance in segregation studies, percentages cannot reflect the two distinct dimensions of segregation (13, 17, 18). From a critical point of view, the continued use of percentages as a “proxy” measure of segregation in health research would obscure the understanding of pathways by which residential segregation may shape health.

As Kramer and Hogue (4) argued, the evenness and isolation dimensions of segregation may have different effects on health; in other words, plausible protective and adverse (or null) effects of segregation may vary by health behaviors (e.g., diet, physical activity, and smoking) and health outcomes (e.g., cancer, obesity, and mortality), as well as by age, gender, race/ethnicity, and other sociodemographic characteristics. However, without incorporating effective and meaningful segregation measures in describing the neighborhood characteristics into health research, only limited (if not biased) knowledge can be gained from future studies. Much effort is, therefore, needed to build upon the conceptual and methodological foundations of measuring segregation established by demographers, geographers, and sociologists. In order to promote informative research, this paper offers several important conceptual remarks about measuring segregation, related methodological challenges, and practical approaches to set the stage for using segregation measurements in future studies. Two Midwestern US cities, St. Louis, MO, USA and Chicago, IL, USA, are used as examples to illustrate these points.

## Materials and Methods

### Data

Population characteristics by race/ethnicity, poverty status, and employment status at the census tract level were obtained from the 2005 to 2009 American Community Survey (ACS) for St. Louis, MO, USA (St. Charles County, St. Louis County, and St. Louis City) and Chicago, IL, USA (Cook County). Census tract data were used partly because both poverty and employment statuses were not available from the 2005 to 2009 ACS at the block group level. In this study, unpopulated census tracts were removed from the analysis. The 2000 US census tract boundary file was obtained from the US Census Bureau. Since census tract boundaries extend into rivers and/or include large ponds and lakes, such water bodies were also removed from the boundary file when the total land area (square kilometer) was recalculated in GIS (ArcGIS 10; ESRI Inc., Redlands, CA, USA). The population and geographic characteristics of these two Midwestern US cities are summarized in Table 1.

**Table 1:** **Description of the two Midwestern US cities**.

		St. Louis, MO, USA	Chicago, IL, USA
Total land area^a^	(km^2^)	2,918	2,433
Number of census tract^b^		340	1,327
Total population^b^		1,692,563	5,257,001
Non-Hispanic White^b^	(%)	70.0	45.2
Non-Hispanic Black^b^	(%)	23.3	25.3
Hispanic^b^	(%)	2.3	22.5
Other racial/ethnic groups^b^	(%)	4.4	7.0
Below poverty^b^	(%)	11.2	14.9
Unemployed^b^	(%)	7.2	9.3

*^a^Derived from the GIS recalculation (not including bodies of water) by the authors*.

*^b^Derived from the 2005 to 2009 American Community Survey (ACS)*.

### Local spatial segregation measures

Traditional segregation measures are aspatial, meaning that when the populations of any two areal units are swapped, the segregation level of the entire region reflected by these aspatial measures remain unchanged. That is, these measures fail to consider population characteristics in the surrounding neighborhoods. Local segregation measures, which provide a segregation value for each local unit within a region, share the same deficiency that it is independent of the population characteristics in neighboring units. By incorporating the concept of modeling local spatial autocorrelation (19, 20), Wong (15) suggested using the function *c_ij_*(.) to modify aspatial segregation indexes into spatial segregation indexes. In spatial autocorrelation studies, *c_ij_*(.) is the element of a binary matrix where “1” indicates areal units *i* and *j* are neighbors, and “0” otherwise. Different from the *c_ij_*(.) typically used in spatial autocorrelation studies, *i* can equal to *j* and thus *c_ii_* = 1. Therefore, integrating the function *c_ij_*(.) with some segregation indexes provides a means to include populations in neighboring units that can account for the potential spatial interaction of population groups across unit boundaries. This is the concept of composite population (15). For example, the composite population count of group G in areal unit *i* (*cg_i_*) is modeled as

$$cg_{i}=\sum_{j}c_{ij}g_{j}$$

where *g_j_* is the population count of group G in areal unit *j*. In other words, a composite population count refers to the population count in areal unit *i* plus the population counts in its neighboring units *j*.

The general concept of composite population count is to include the population of neighboring units in evaluating the population of a reference unit. The specific version of *c_ij_* in the initial implementation of composite population counts was a binary function of neighbors such that the population of a neighboring unit is included or not. Feitosa et al. (8) used a kernel function to derive the local population intensity, the same concept as the composite population count such that populations farther away are weighted less, while closer are weighted more. To a large degree, this kernel estimator captures the distance decay effect commonly considered in geographical research, reflecting the general fact that interaction declines with increasing distances. However, this formulation is slightly different from the “population *density* of the local environment” suggested by Reardon and O’Sullivan (17). Their function used a spatial *density* kernel to enumerate the population count of an areal unit, but these “(w)eighted density values depend on the spatial arrangement (distance between cells) and on the areas of the spatial units (cells),” and therefore, it is more affected by the sizes of areal units when spatially aggregated data are used (8).

While these subsequent developments employed more elegant spatial weighting schemes, but are more difficult to implement than the binary weights in the composite population counts, the importance is the notion that enumeration unit boundaries do not delimit the spatial interaction of population groups, and neighboring populations need to be considered in measuring the levels of segregation. Based upon the concept of composite population counts, Wong (9, 10) introduced a family of local spatial segregation indexes: the local spatial dissimilarity index (*SD_i_*), the local spatial entropy-based diversity index (*SH_i_*), and the local spatial isolation index (*S_i_*). Massey and Denton (13) claimed that the traditional dissimilarity index (*D*) is best to capture the evenness dimension, while Reardon and Firebaugh (34) advocated the use of diversity index (*H*). *H* is another measure for the evenness dimension, which is also commonly referred to as the Shannon index in biology and ecology. Lieberson’s isolation index (P*) has been regarded as the standard for the isolation dimension (35). By definition, Wong’s indexes are the local spatial versions of these global aspatial indexes, corresponding to the two distinct dimensions of segregation (i.e., evenness and isolation). These specifications are given as follows:

White–black dissimilarity (SD_i*wb_)

(1) $$SD_{i\text{*}wb}=\left|\frac{cw_{i}}{CW}-\frac{cb_{i}}{CB}\right|$$

where *cw_i_* and *cb_i_* are the composite population counts of whites and blacks in areal unit *i*, respectively, and *CW* and *CB* are the composite population counts of whites and blacks for the entire study area, respectively. This is the local spatial version of the popular *D*.

White–others dissimilarity (SD_i*wo_)

(2) $$SD_{i\text{*}wo}=\left|\frac{cw_{i}}{CW}-\left(\frac{ct_{i}-cw_{i}}{CT-CW}\right)\right|$$

Black–others dissimilarity (SD_i__*bo_)

(3) $$SD_{i\text{*}bo}=\left|\frac{cb_{i}}{CB}-\left(\frac{ct_{i}-cb_{i}}{CT-CB}\right)\right|$$

where *ct_i_* is the composite population count of total population in areal unit *i*, and *CT* is the composite population count of total population for the entire study area. Whereas Eq. 1 compares the distributions of two groups, Eqs 2 and 3 compare one group with the remainder of the population. These are also the local spatial versions of the popular *D*.

Racial/ethnic diversity (*SH_i_*)

(4) $$SH_{i}=-\sum_{k}^{n}\left(\frac{cp_{ik}}{ct_{i}}\right)\ln\left(\frac{cp_{ik}}{ct_{i}}\right)$$

where *cp*_*ik*_ is the composite population count of mutually exclusive group *k* in areal unit *i* (e.g., whites, blacks, Hispanics, … *n*). This is the local spatial version of the popular *H*.

White isolation (S_i__*wo_)

(5) $$S_{i\text{*}wo}=1-\left(\frac{ct_{i}-cw_{i}}{T-W}\right)$$

Black isolation (S_i__*bo_)

(6) $$S_{i\text{*}bo}=1-\left(\frac{ct_{i}-cb_{i}}{T-B}\right)$$

where *W, B*, and *T* are the population counts of whites, blacks, and total population for the entire study area, respectively. Here, the original formulations (9) were simplified in this study by replacing the neighborhood operators with the composite population counts. These are the modified local spatial versions of the popular P*.

Note that either equations 5 or 6 can be applied to model the spatial interaction of below and above the poverty status as poverty isolation, as well as of unemployed and employed (among the civilian non-institutional population aged 16 years and older) status as unemployment isolation.

These local spatial segregation measures were computed in R (36). The local spatial dissimilarity measures (derived from *SD_i_*) and the local spatial entropy-based diversity measures (derived from *SH_i_*) were standardized by their maximum values, while the local spatial isolation measures (derived from *S_i_*) were standardized by their range, such that all measures are bounded between 0 and 1. These standardizations are justifiable as the purpose of this study is to examine intra-urban variations, not inter-city differences.

### Analysis

**Figure 2:**
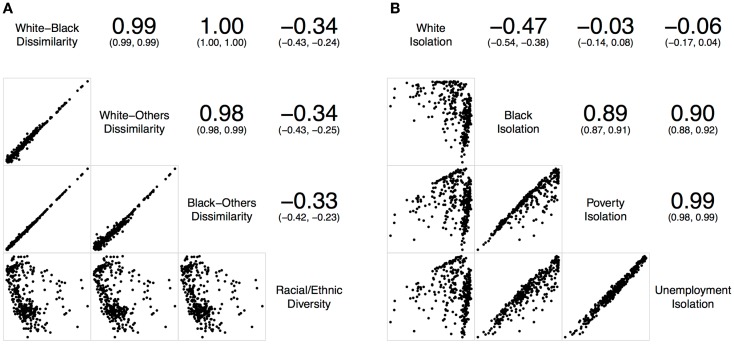
**Correlations of local spatial segregation measures in St. Louis, MO, USA (340 census tracts)**. **(A)** With respect to the evenness (i.e., dissimilarity and diversity) dimension, and **(B)** with respect to the isolation dimension.

**Figure 3:**
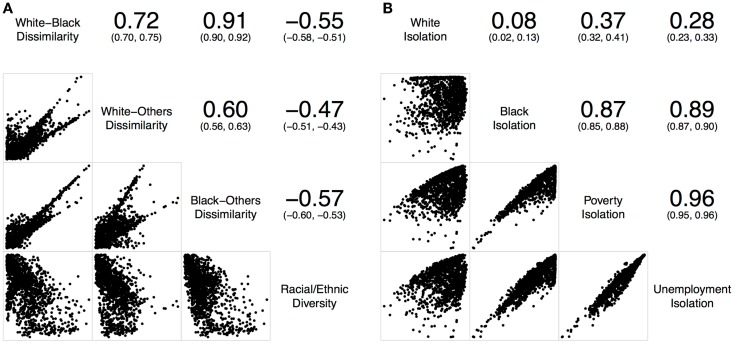
**Correlations of local spatial segregation measures in Chicago, IL, USA (1,327 census tracts)**. **(A)** With respect to the evenness (i.e., dissimilarity and diversity) dimension, and **(B)** with respect to the isolation dimension.

**Figure 4:**
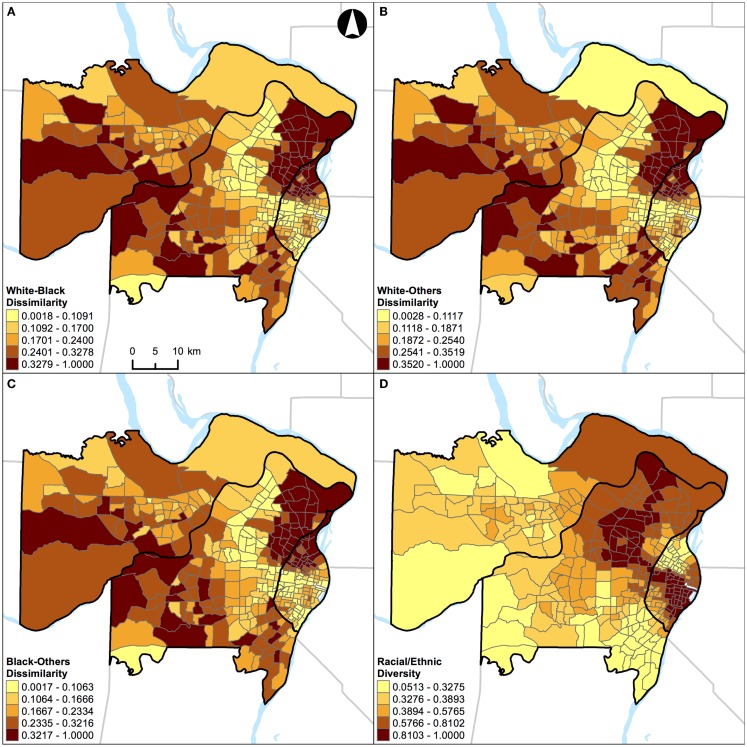
**Geographic distribution of local spatial evenness measures in St. Louis, MO, USA (340 census tracts)**. **(A)** White–black dissimilarity, **(B)** white–others dissimilarity, **(C)** black–others dissimilarity, and **(D)** racial/ethnic diversity. A quantile classification scheme was used to display the levels of local segregation.

**Figure 5:**
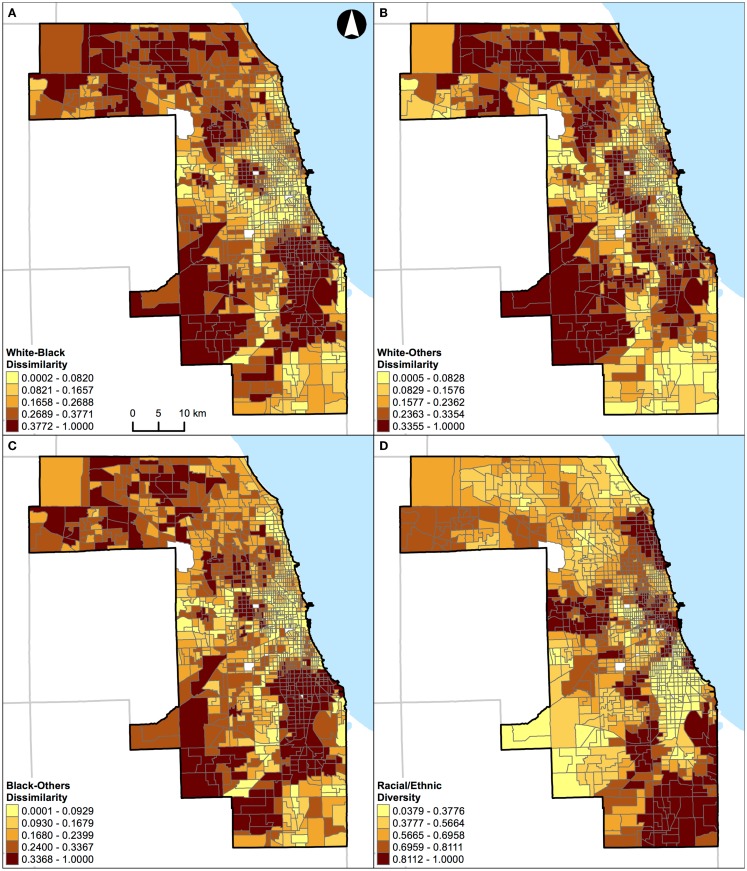
**Geographic distribution of local spatial evenness measures in Chicago, IL, USA (1,327 census tracts)**. **(A)** White–black dissimilarity, **(B)** white–others dissimilarity, **(C)** black–others dissimilarity, and **(D)** racial/ethnic diversity. A quantile classification scheme was used to display the levels of local segregation.

**Figure 6:**
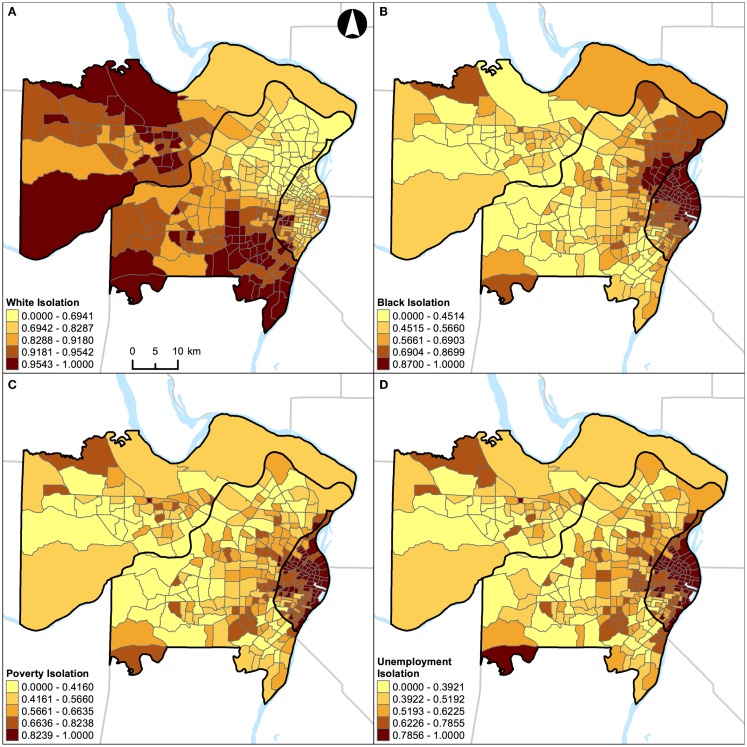
**Geographic distribution of local spatial isolation measures in St. Louis, MO, USA (340 census tracts)**. **(A)** White isolation, **(B)** black isolation, **(C)** poverty isolation, and **(D)** unemployment isolation. A quantile classification scheme was used to display the levels of local segregation.

**Figure 7:**
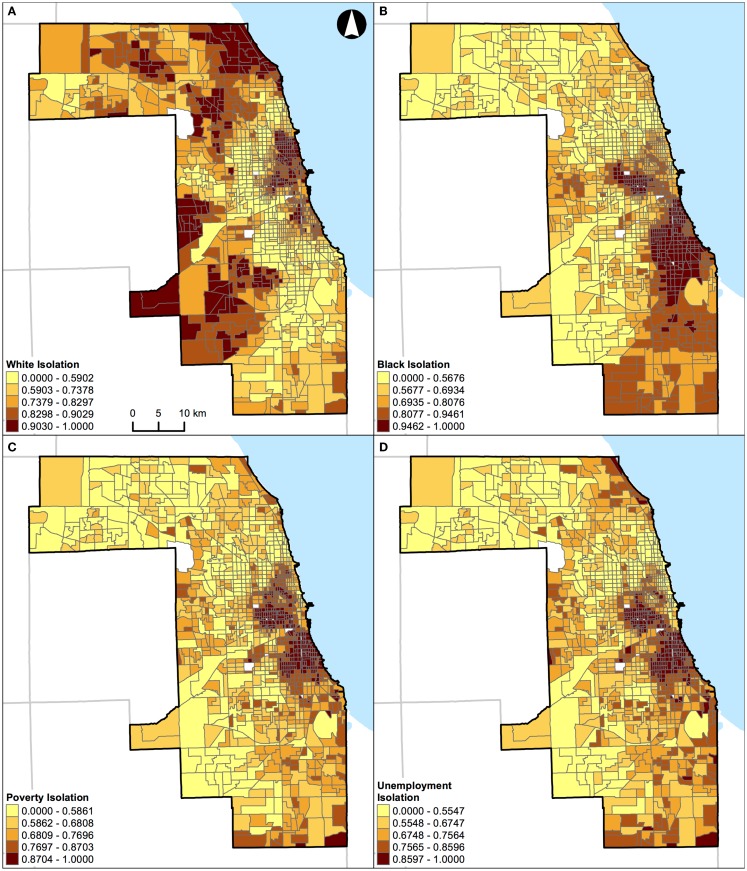
**Geographic distribution of local spatial isolation measures in Chicago, IL, USA (1,327 census tracts)**. **(A)** White isolation, **(B)** black isolation, **(C)** poverty isolation, and **(D)** unemployment isolation. A quantile classification scheme was used to display the levels of local segregation.

To examine the relationships of local spatial segregation measures derived from above, a series of correlation statistics (37) were conducted in R (38) for St. Louis, MO, USA (Figure 2) and Chicago, IL, USA (Figure 3). Correlations and scatterplot matrixes were used to display the relationships. The upper off-diagonal panels show the correlation coefficients with associated 95% confidence intervals (in parentheses), and the lower off-diagonal panels show the scatter plots. The geographical distributions of local spatial segregation measures with respect to the evenness (Figures 4 and 5) and isolation (Figures 6 and 7) dimensions within the two Midwestern US cities are shown in maps. A quantile classification scheme was used to display the levels of local segregation.

**Figure 8:**
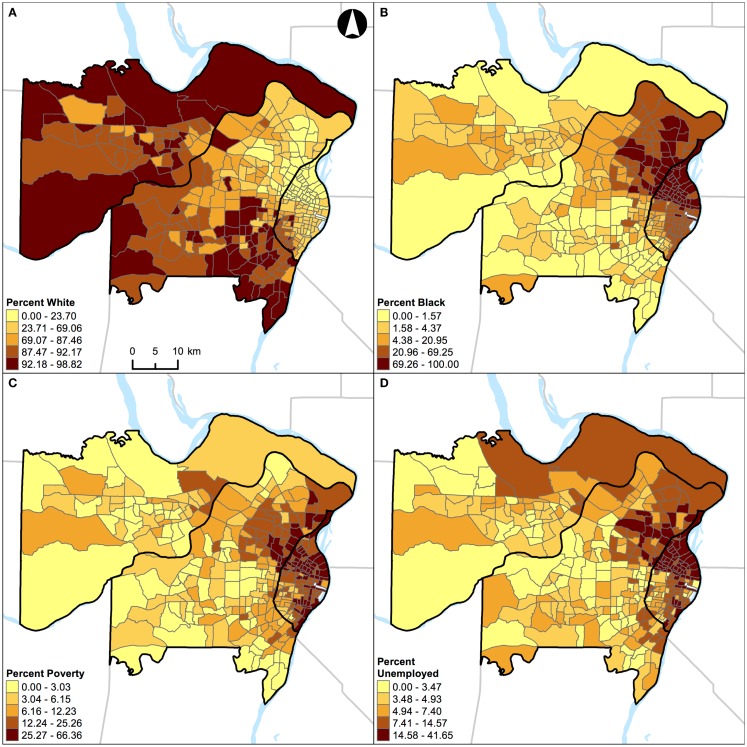
**Geographic distribution of population characteristics expressed as percentages in St. Louis, MO, USA (340 census tracts)**. **(A)** Percent white, **(B)** percent black, **(C)** percent Hispanic, and **(D)** percent other racial/ethnic groups. A quantile classification scheme was used to display the percentages.

**Figure 9:**
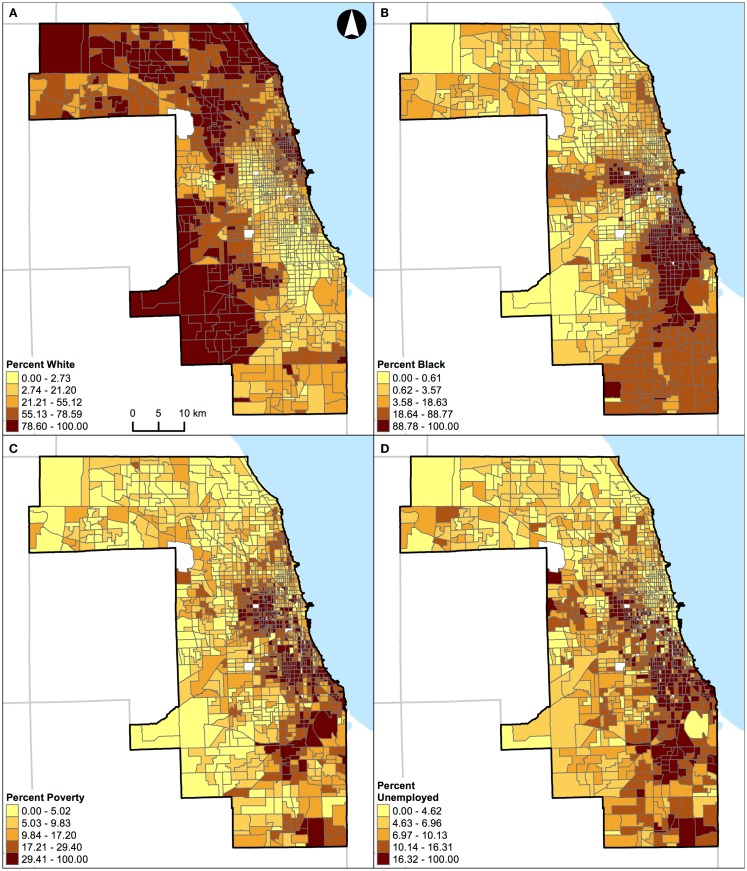
**Geographic distribution of population characteristics expressed as percentages in Chicago, IL, USA (1,327 census tracts)**. **(A)** Percent white, **(B)** percent black, **(C)** percent Hispanic, and **(D)** percent other racial/ethnic groups. A quantile classification scheme was used to display the percentages.

For demonstration purposes, the geographical distributions of percent white, black, Hispanic, and other racial/ethnic groups are also shown for St. Louis, MO, USA (Figure 8) and Chicago, IL, USA (Figure 9).

## Results

Table 1 shows the geographic and population characteristics of the study area. In brief, St. Louis, MO, USA and Chicago, IL, USA are in the same geographic region with a similar total land area. However, these two Midwestern US cities were different in terms of their population sizes, number of census tracts, and racial/ethnic compositions. In addition, the poverty and unemployment rates were slightly higher in Chicago, IL, USA than those in St. Louis, MO, USA.

Figure 2A shows the correlations of different local spatial segregation measures with respect to the evenness (i.e., dissimilarity and diversity) dimension in St. Louis, MO, USA. The white–black, white–others, and black–others dissimilarity measures were highly and positively correlated with one another (0.98 ≤ *r* ≤ 1.00). However, these dissimilarity measures were not correlated with the racial/ethnic diversity measure (−0.33 ≤ *r* ≤ −0.34). On the other hand, Figure 2B shows the correlations of different local spatial segregation measures with respect to the isolation dimension. While the white isolation measure was moderately, but negatively correlated with the black isolation measure (*r* = −0.47), it was not correlated with either the poverty (*r* = −0.03) or unemployment (*r* = −0.06) isolation measures. However, the black, poverty, and unemployment isolation measures were highly and positively correlated with one another (0.89 ≤ *r* ≤ 0.99).

Similarly, Figure 3A shows the correlations of different local spatial segregation measures with respect to the evenness (i.e., dissimilarity and diversity) dimension in Chicago, IL, USA. The white–black dissimilarity measure was moderately and positively correlated with the white–others (*r* = 0.72) and highly and positively correlated with the black–others (*r* = 0.91) dissimilarity measures where the white–others dissimilarity measure was moderately correlated with the black–others dissimilarity measure (*r* = 0.60). In addition, these dissimilarity measures were moderately and negatively correlated with the racial/ethnic diversity measure (−0.47 ≤ *r* ≤ −0.57). On the other hand, Figure 3B shows the correlations of different local spatial segregation measures with respect to the isolation dimension. The white isolation measure was not correlated with the black (*r* = 0.08), poverty (*r* = 0.37), or unemployment (*r* = 0.28) isolation measures. However, the black, poverty, and unemployment isolation measures were highly and positively correlated with one another (0.87 ≤ *r* ≤ 0.96).

Taken together, Figures 2 and 3 consistently show that: (i) the two-group-based dissimilarity measures do not capture the local variation of racial/ethnic segregation as the multiple-group-based diversity measure does, (ii) white isolation was neither equal to nor the inverse of black isolation, and (iii) black isolation occurred conjointly with the poverty and unemployment isolation. However, it is important to note that Figures 2 and 3 also show slightly inconsistent degrees of correlation. In general, St. Louis, MO, USA has higher correlations among the dissimilarity measures than those in Chicago, IL, USA, but their correlations with the racial/ethnic diversity measure were lower in St. Louis, MO, USA than those in Chicago, IL, USA (Figure 2A versus Figure 3A). In addition, St. Louis, MO, USA has higher, but modest, correlations between the white and black isolation measures than those in Chicago, IL, USA. However, the correlations among the white, poverty, and unemployment isolation measures were higher in Chicago, IL, USA than those in St. Louis, MO, USA (Figure 2B versus Figure 3B). These inconsistent degrees of correlation between the two Midwestern US cities were primarily due to the difference in racial/ethnic compositions (Table 1); the larger proportions of Hispanic and other racial/ethnic groups in Chicago, IL, USA contributes more to the spatial patterns than those in St. Louis, MO, USA.

Results of correlation analysis in Figures 2A and 3A are also manifested spatially. Figures 4 and 5 show the geographic distributions of local spatial evenness (i.e., dissimilarity and diversity) measures in St. Louis, MO, USA and Chicago, IL, USA, respectively. In Figures 4A–C, the dissimilarity measures between white–black, white–others, and black–others exhibit very similar spatial patterns in St. Louis, MO, USA, with clusters of high segregation in the city center (downtown) and patches in the western outskirts. The high degrees of concurrences between these dissimilarity measures were primarily driven by the spatial pattern of blacks as they constitute the dominant minority group in the region. However, such high degrees of resemblance between these dissimilarity measures were not found in Chicago, IL, USA (Figures 5A–C). Differences between these two Midwestern US cities are visually recognizable, as besides whites and blacks, Hispanics population constitutes a major proportion in Chicago, IL, USA (Table 1). As pointed out above in the correlation analysis, the dissimilarity and diversity measures do not have an inverse relation (Figures 2A and 3A). By comparing Figures 4D and 5D with Figures 4A–C and 5A–C, respectively, they confirm that the racial/ethnic diversity measure captures more than just the opposite of dissimilarity; areas with the highest (or lowest) values in Figures 4A–C and 5A–C do not always have the lowest (or highest) values in Figures 4D and 5D, respectively.

In addition, results of correlation analysis in Figures 2B and 3B are also manifested spatially. Figures 6 and 7 show the geographic distributions of local spatial isolation measures in St. Louis, MO, USA and Chicago, IL, USA, respectively. Figures 6B–D and 7B–D exhibit very similar spatial patterns thereby reflecting the fact that blacks are the socioeconomically disadvantaged group in these two Midwestern US cities. Although the spatial patterns of white isolation (Figures 6A and 7A) are different from those of the black, poverty, and unemployment isolation (Figures 6B–D and 7B–D, respectively), they are not reversed images of each other. In fact, some areas, such as the southwestern and northwestern corners of St. Louis, MO, USA seem to have relatively high isolation levels in all situations (Figures 6A–D). On the other hand, in the northeastern corner of Chicago, IL, USA, it has both relatively high levels of white and unemployment isolation (Figures 7A,D). In order to explain these spatial patterns and their relationships, further research is needed to understand in detail the interaction between racial/ethnic and socioeconomic segregations. However, it is beyond the scope of this study. When data about health are brought into the analysis, Figure 4 through Figure 7 can serve as the foundations for exploratory spatial data analysis (ESDA) to assist the formulations of hypotheses (39, 40) in examining the relationships between residential segregation and health.

Unlike the segregation measures described above, percentage of population belonging to a group (e.g., percent black) cannot be used as a “proxy” measure of segregation (33) in future research. This argument can be illustrated by examining the geographic distributions of percent white, black, Hispanic, and other racial/ethnic groups in the two Midwestern US cities (Figures 8 and 9). For example, in St. Louis, MO, USA, a lower percentage of white (Figure 8A) corresponds to higher percentages of other population groups in different parts of the region: black in the northeastern parts (Figure 8B), Hispanic in the north central and southeastern parts (Figure 8C), and other racial/ethnic groups in the central parts (Figure 8D). In addition, higher percentages of black (Figure 8B), Hispanic (Figure 8C), and other racial/ethnic groups (Figure 8D) co-occur in the north central and southeastern parts of St. Louis, MO, USA. Similarly, in Chicago, IL, USA, a lower percentage of white (Figure 9A) corresponds to higher percentages of other population groups in different parts of the region: black in the central and southeastern parts (Figure 9B), Hispanic in the northern, central, and southeastern parts (Figure 9C), and other racial/ethnic groups along the shore of Lake Michigan and in the northern parts (Figure 9D). In addition, higher percentages of white (Figure 9A), black (Figure 9B), Hispanic (Figure 9C), and other racial/ethnic groups (Figure 9D) co-occur along the shore of Lake Michigan as well as in the central and southern parts of Chicago, IL, USA. If percentages were used to measure the level of segregation, such usages would suggest that: (i) a lower percentage of white would refer not only to a higher percentage of black (i.e., the inverse thereof) but also to higher percentages of Hispanic and other racial/ethnic groups, but these high percentages may not be true, and (ii) a higher percentage of a racial/ethnic group (particularly among the minority groups) would refer to both a racially/ethnically dominated and integrated neighborhood. Taken together, Figures 8 and 9 corroborate the fact that percentages cannot reflect the two distinct dimensions of segregation (13, 17, 18).

One of the central principles in segregation studies is that measures need to quantify how different population groups are distributed across areal units. Putting aside the aspatial nature for a moment, the segregation indexes have been developed by demographers, geographers, and sociologists to measure the extent to which two or more groups are distributed across areal units within a given region. Simple percentages cannot capture the between-unit relationship as implicitly accounted for in most segregation measures, aspatial or spatial indexes alike. For these reasons, using local spatial segregation indexes to reflect the evenness and isolation dimensions are preferable in future studies.

## Discussion

In this study, St. Louis, MO, USA and Chicago, IL, USA were examined. They are in the same geographical region with similar total land areas, but different population characteristics (Table 1). As pointed out above, the correlations of local spatial segregation measures showed consistent patterns in these two Midwestern US cities (Figures 2 and 3), and these correlations were also manifested spatially (Figure 4 through Figure 7). As a practical guide to segregation studies, Figures 8 and 9 demonstrated that percentages cannot be used as a measure of segregation. These results, in turn, highlight two important remarks about the use of local spatial segregation indexes in health research.

First, the use of local spatial entropy-based diversity index (*SH_i_*) is recommended for measuring the local variation of racial/ethnic segregation with respect to the evenness dimension. Both the dissimilarity and diversity indexes have been classified as measures of evenness among the dimensions of segregation (13, 17). From a conceptual point of view, these two measures are the inverse of each other. As shown in Figures 2A, 3A, 4, and 5, however, such an expectation does not generally hold. By and large, the dissimilarity index (*D*) has become one of the most popular (if not considered to be the standard) measure of segregation. Nevertheless, the use of *D* in segregation studies has long been criticized for its limitations [e.g., Ref. (41–43)] since it was first introduced nearly six decades ago by Duncan and Duncan (44). In fact, Cortese et al. (45) demonstrated some of the systematic biases in *D* more than three decades ago. Among existing measures of segregation, the entropy-based diversity index (*H*) has been determined as a superior measure of the evenness dimension (34, 46). Because *H* is aspatial in nature, the use of its spatial version (*SH*) is logical in segregation studies (9, 10, 17). In a world of increasing globalization, the US has become markedly more racially and ethnically diverse (mainly, due to the increases in Hispanic and Asian populations) during the recent decades (47). Unlike the past, the situations of multiple racial/ethnic groups are the norms rather than the exception (15, 34). Future research on the relationship between residential segregation and health, therefore, should consider using *SH_i_* to measure the evenness dimension of racial/ethnic segregation in order to account for the spatial dimension and the multiracial and ethnic nature of the contemporary US society.

Second, the use of local spatial isolation index (*S_i_*) is recommended not only for measuring the local variation of racial/ethnic segregation but also for socioeconomic (e.g., poverty and unemployment) segregation with respect to the isolation dimension. From a structural view of racial phenomena in the US, segregation of blacks has been considered as the institutional manifestations of racism: the racial organization of the society is structured by the underlying psychological, cultural, social, economic, and political phenomena over time. In other words, the observed racial disparity is induced and maintained because a society possesses a racialized social system that determines the relationship and interaction between races (48). Particularly for blacks, limited educational and employment opportunities, redlining and housing discrimination, and adverse psychosocial distress are some of the societal factors coupled with residential segregation (2, 3). Due to the multifaceted nature of racial phenomena, segregation of blacks may ultimately constrain their wealth accumulation and upward social mobility. At the national level, poor blacks have been segregated from other racial/ethnic and income groups, and the magnitude of segregation had weakened only slightly between 1970 and 2000 (49). Overall, a major shift of blacks into less-segregated (or integrated) areas has not occurred (50). Taken together, these historic and societal factors are likely to play a role in constraining poor and unemployed blacks to live in certain neighborhoods, but not for whites (Figures 2B, 3B, 6, and 7). Future research on the relationship between residential segregation and health, therefore, should consider using *S_i_* to measure the isolation dimension of racial/ethnic and socioeconomic segregations to account for the spatial dimension and the multifaceted nature of population distribution in the US.

Despite the number of studies conducted to date (4), the wide adoptions of ineffective and insufficient segregation measures reflect the ongoing trend that the methodological advancements in segregation studies achieved by demographers, geographers, and sociologists have not been adequately translated into health research. Namely, the percentage of population belonging to a group (e.g., percent black) has been widely used as a measure of segregation in most previous studies based on a local approach. To our knowledge, there has not been a study that used the local spatial segregation indexes for measuring racial/ethnic diversity and socioeconomic (e.g., poverty and unemployment) isolation in health research; only four studies used *S_i_* for measuring black isolation in a form of continuous and log-transformed variables (27, 51) or dichotomous categorical variables (28, 52). As a supplemental note, two studies incorporated the segregation indexes based on spatial kernels (17): black–others dissimilarity and black isolation as continuous and binary variables (53), and black isolation as a categorical variable (54). However, no study has incorporated the segregation indexes developed by Feitosa et al. (8). Notwithstanding the methodological differences (as noted earlier), the segregation indexes developed by Reardon and O’Sullivan (17) and Feitosa et al. (8) are comparable to, but methodologically more elegant than those developed by Wong (9, 10).

Toward the use of *SH_i_* and *S_i_* as covariates in health research, however, there are technical and theoretical challenges that need to be explored in future studies, especially in the context of linear regression analysis. That is, these two local spatial segregation measures do not necessarily follow a normal distribution and may be skewed or highly skewed. In some instances, neither applying the traditional transformations (e.g., square root, log, and inverse) nor the Box–Cox (i.e., parametric power) transformation (55) can achieve normality. Even when normality has been achieved, data transformations can introduce difficulties in interpreting the results as they alter the nature of the variable. As a means to handle skewed or highly skewed distributions, continuous variables have commonly been categorized into quantiles (most often tertiles or quartiles) based on various approaches [e.g., Ref. (56–61)]. Dichotomizing continuous variables has been widely regarded as an inappropriate practice that generates rather than solves problems (62, 63). However, it is equally important to recognize that categorization of continuous variables has also been criticized for its unrealistic assumption of homogeneity within categories, and its rather arbitrary data-driven cut points used to define categories that may create difficulties to compare results across studies (64, 65). To date, there is no theoretical or empirical basis for determining the optimal cut points in categorizing the local spatial segregation measures.

Further research may be needed to determine thresholds for which the local spatial segregation measures have protective or adverse effects on health. However, it may not be feasible to identify the threshold levels that apply to different localities, particularly for the evenness dimension. For example, a growing racial/ethnic diversity related to the increases in Hispanic and Asian populations varies from region to region in the US (47). Also, within-city and within-suburban sorting of racial/ethnic populations does not occur evenly or uniformly across the US (66). Even between St. Louis, MO, USA and Chicago, IL, USA, which are roughly 400 km apart, their racial/ethnic compositions are quite different (Table 1); in particular, the proportion of Hispanics in St. Louis, MO, USA is much lower than that in Chicago, IL, USA. As a result, the increase of racial/ethnic diversity (derived from *SH_i_*) would mostly reflect white–black (i.e., racially) integrated neighborhoods in St. Louis, MO, USA, whereas such an increase would reflect white–black–Hispanic (i.e., racially and ethnically) integrated neighborhoods in Chicago, IL, USA. This, in turn, suggests that the potential interaction of racial/ethnic groups and thus their experience in social environments are not alike; the same level of racial/ethnic diversity measure across cities does not necessarily mean that the same degree and nature of racial/ethnic diversity are present. For these reasons, the threshold effects of local spatial segregation on health should be explored with careful consideration and clear justifications. In health research, generalizability and transportability are two important components that should be kept in mind.

Another challenge of using *SH_i_* and *S_i_* in a regression framework is related to the processes of how they are computed. Regardless of whether they are derived using the simplistic composite population concept (9, 10) or the elegant spatial kernels (8, 17), the basic principle of local spatial segregation indexes is to remove the enumeration boundaries as the absolute barriers to inter-group interaction by aggregating populations across adjacent or contiguous neighborhoods. As noted earlier, this is a more realistic portrayal of the social interaction among neighbors in our daily lives than that of such interaction to occur only within the confined unit boundary. The aggregation processes smooth the distribution of population spatially by which the smoothed data lead to a better detection of the systematic trends in a given area. However, such aggregation processes can also magnify the level of spatial autocorrelation (i.e., residuals that vary systematically over space), which violates the independence assumption. For example, LeSage (67) demonstrated how ignoring the spatial configuration of geographic data in a linear regression model can produce unstable parameter estimates and yield unreliable statistical significance testing results. Although a certain level of spatial autocorrelation is expected, particularly when the data are from an urban context, both *SH_i_* and *S_i_* are likely to accentuate the magnitude and structure of positive spatial autocorrelation. Different types of spatial regression models have been introduced to account for spatial dependencies, such as the spatial error or spatial lag models and the eigenvector-based spatial filtering method [e.g., Ref. (68–70)]. Their implementations, nevertheless, create another layer of complexity in statistical modeling. To our knowledge, no study examined the relationships between residential segregation and health using the local spatial segregation measures (derived from *SH_i_* and *S_i_*) in spatial regression models.

These technical and theoretical challenges can restrict the use of *SH_i_* and *S_i_* in a regression model when an outcome of interest is a continuous variable. Without due consideration, the distribution of these two local spatial segregation measures may violate the assumptions of linearity, normality, homoscedasticity, and/or independence. Nonetheless, an outcome of interest is often, or can be classified into, a binary variable in health research (e.g., obesity and smoking status). Unlike a linear regression model, a logistic regression model (as well as other types of generalized linear models) does not require normality in the distributions of covariates and homoscedasticity (i.e., homogeneity of variance), and has less stringent requirements (71). For this reason, as long as the linearity in the logit assumption is satisfied, logistic regression that accounts for spatial autocorrelation can be used to examine the relationships between the local spatial segregation measures (derived from *SH_i_* and *S_i_*) and health-related outcomes. Various spatial logistic models have been developed in recent years, but the results and conclusions may vary between different models [e.g., Ref. (72)]. As a preliminary step for fitting regression models to spatial data, a model selection should be based on the comparative analysis of different models (73).

More appropriately, from a statistical perspective, multilevel logistic models should be used instead of spatial logistic models. Here, multilevel (i.e., hierarchical, mixed, nested, mixed-effect, or random-effect) models refer to regression models that combine traditionally distinct individual and ecological models, and to overcome the limitations in focusing only on one level (74, 75). The use of multilevel models is a necessity in exploring the relationships between residential segregation and health (1) because both individualistic and ecological fallacies can lead to inaccurate (if not misleading) conclusions (76). In order to conduct an informative analysis, covariates at multiple levels should be taken into consideration in explaining health-related outcomes. At least four studies have examined the relationship between residential segregation and health by using the spatial isolation index in multilevel models (27, 28, 51, 54). Hence, the use of multilevel models is recommended as the fundamental statistical approach in future studies.

By taking advantage of modern computational and statistical methods, a more sophisticated approach to commonly used multilevel models is the use of (Bayesian) generalized additive mixed models [e.g., Ref. (77)]. Given the complexity of such statistical models (77), however, they must be considered with care.

## Limitations

Despite the potential importance of using *SH_i_* and *S_i_* in health research as illustrated above, these two local spatial segregation indexes share common concerns with other spatial analytical techniques and segregation studies. Among them, two inherent limitations warrant mentioning. For one, both *SH_i_* and *S_i_* are subject to the boundary or edge effect, which is typically unavoidable when using areal and geographic data. Such an effect introduces bias into the identification of spatial distribution and the parameter estimates of spatial process (78). That is, in almost any given geographical and health studies, a boundary has to be demarcated as the study area: a city, a metropolitan area, or a region defined by one or more counties in the US. While only subunits within the study area are the concerns in the analysis, units outside of the demarcated boundary would be ignored completely. In other words, areal units within the study areas are clipped from the rest of the geography to form an island, isolated from the rest in the analytical setting. The exception is in a unique setting where the study area is entirely surrounded by a large body of water (e.g., studies conducted within the Hawaii islands). Since areal association, geographic distribution, and spatial interaction extend beyond the demarcated boundary, when the study area is “lifted” from its surrounding geography in the analysis, the measures or statistics computed would be biased. In fact, all units within the entire study area would be affected, but the effects are stronger for areal units closer to the border than those closer to the center of the study area. Inevitably, the local spatial segregation measures (*SH_i_* and *S_i_*) would be affected by the boundary or edge effect.

Several solutions have been proposed to address the boundary or edge effect during the past decades. Nonetheless, all of them have misgivings, and they cannot be implemented easily [e.g., Ref. (79)], or can they fully solve the problem. One rather simple approach, which was not implemented in this study, is to include a buffer zone around the study area. For instance, additional areas to the west of the two Midwestern US cities can be used to create the buffer zone. However, the Mississippi River, which runs along the east side of St. Louis, MO, USA, physically separates the city from East St. Louis, IL, USA, another city to the east. While a couple of bridges and public transportation systems connect the Missouri and Illinois sides, limited transit options to cross the river substantially reduce the spatial interaction of population groups between the two sides of the river. In the computation of local spatial segregation measures using *SH_i_* and *S_i_*, all areal units are treated as spatially continuous regardless of such boundary lines being the street or river. In reality, however, the spatial interaction of population groups and their experience in the social environments are not the same for people crossing the street versus the river. Given the physical separation between the two sides of the river, it may not be appropriate to extend a buffer zone beyond the Mississippi River. Otherwise, a careful consideration is needed for creating a buffer zone into the Illinois side. Unlike St. Louis, MO, USA, Lake Michigan lies on the east side of Chicago, IL, USA, and thus, creating a buffer zone will not be appropriate and feasible.

In general, the larger the buffer zone, the less will be the boundary or edge effect. The ideal size of a buffer zone is to ensure that all areal units within the study area are not affected. However, such a size is very difficult to determine; it will be partly dependent upon the spatial analytical technique adopted and the data used in a study, which may or may not be place specific. Because a gold standard does not exist, a very careful consideration is needed instead in handling areal and geographic data (80, 81). One simple guideline is to identify the nature of the spatial relationship to be involved, and then to determine the size of the buffer zone in minimizing the boundary or edge effect to an acceptable level. In the context of *SH_i_* and *S_i_*, the function *c_ij_*(.) adopted to implement the concept of composite population involves only the immediate neighboring units (15). For practical purposes, therefore, a buffer zone including the first-order adjacent units along the study area will be sufficient for using these two local spatial segregation indexes to measure the levels of segregation.

Lastly, a local approach (and in fact to a large degree, a global approach) to measure socioeconomic (e.g., poverty and unemployment) segregation would encounter a challenge in terms of data quality. In particular, this issue pertains to the US situation. After 2000, US Census no longer gathers detailed information on household’s socioeconomic status and living conditions through the so-called long form in previous decennial censuses. Replacing the long form is the ACS, which is a continuous measurement program surveying US population, and is the major source of socioeconomic and housing data. Due to the nature and design of ACS, however, its estimates for smaller geographical units (e.g., census tracts and block groups) may not be reliable (with relatively large margin of errors) as compared with data from past decennial censuses. Nevertheless, how the quality of ACS estimates may affect the computation of segregation measures in general, and *SH_i_* and *S_i_* in specific have not been investigated. As is to be expected, a curtain of uncertainty will be casted over the socioeconomic segregation measures when the ACS data are used in future studies.

## Conclusion

Segregation is the extent to which individuals of various groups occupy and experience different social environments. As the condition involves more than one group, measuring the levels of segregation needs to account for the spatial interaction of different population groups. Otherwise, segregation measures without accounting for spatial relationships would leave out the essence of segregation. Unlike the global segregation measures that overlook the important variations at the local level, local segregation measures draw attentions to the situations at the neighborhood scale. In particular, two local spatial segregation indexes highlighted in this paper provide effective and meaningful measurements of the two distinct dimensions of segregation: (i) the local spatial entropy-based diversity index (*SH_i_*) for the evenness dimension, and (ii) the local spatial isolation index (*S_i_*) for the isolation dimension. From an analytical point of view, the use of *SH_i_* will help elucidate the relationship between racial/ethnic integration (or, its counterpart, racial/ethnic similarity) and health, whereas the use of *S_i_* will help elucidate the relationships between racial/ethnic and/or socioeconomic isolation and health. These two local spatial segregation indexes can be used in ESDA, assisting the formulations of hypotheses to be tested, and examining the relationship between residential segregation and health. However, they have rarely been incorporated into health research. Hence, future studies should explore the use of *SH_i_* and *S_i_* to better understand both the protective and adverse effects of residential segregation on health.

## Author Contributions

Masayoshi Oka conceptualized the study, obtained the data, carried out the analyses, and drafted the manuscript. David W. S. Wong contributed to the conceptualization of the study, drafted some subsections, and revised the manuscript. All authors read and approved the final manuscript.

## Conflict of Interest Statement

The authors declare that the research was conducted in the absence of any commercial or financial relationships that could be construed as a potential conflict of interest.
